# Toward Precision Fluid Management in Hemodialysis: Adaptive Ultrafiltration Guided by Continuous Relative Blood Volume Monitoring

**DOI:** 10.3390/jcm15187078

**Published:** 2026-09-12

**Authors:** Doris H. Fuertinger, Felix J. Meigel, Aiyuan Wang, Sabrina Casper, Sheng-Han Yueh, Kevin Ho

**Affiliations:** 1 Renal Research Institute LLC, New York, NY 10065, USA; felix.meigel@rriny.com (F.J.M.); sabrina.casper@rriny.com (S.C.); sheng-han.yueh@rriny.com (S.-H.Y.); 2 Fresenius Medical Care North America, Waltham, MA 02451, USA; aiyuan.wang@freseniusmedicalcare.com (A.W.); kevin.ho@freseniusmedicalcare.com (K.H.)

**Keywords:** hemodialysis, ultrafiltration, relative blood volume, blood volume monitoring, fluid management, closed-loop control, physiological feedback, in silico simulation

## Abstract

**Background**: Ultrafiltration (UF) during hemodialysis is typically prescribed as a fixed treatment goal and delivered using a largely constant ultrafiltration rate throughout the session. We evaluated whether Adaptive Ultrafiltration (Adaptive UF), a physiological closed-loop control strategy guided by continuous relative blood volume (RBV) monitoring, could improve RBV target attainment while individualizing fluid removal within predefined treatment constraints. **Methods**: We performed a paired patient-specific in silico study using 119,472 historical hemodialysis treatments from 11,246 U.S. patients. Treatment-specific plasma refill rate profiles were derived from recorded RBV and ultrafiltration data. For each historical treatment, a corresponding Adaptive UF treatment was simulated and compared with the corresponding originally delivered treatment. Outcomes included the percentage of time within a predefined favorable RBV target region, UF delivery, and operational performance. **Results**: Mean time within the RBV target region increased from 36.3% with historically delivered treatments to 56.4% with Adaptive UF, showing an absolute improvement of 20.2 percentage points (95% CI, 19.9–20.5); target attainment improved in 90.0% of patients. Mean area outside the target range decreased from 280.7 to 139.5 RBV %–minutes. Mean achieved UF volume increased by 296.4 mL, although treatment-level UF delivery was bidirectional, increasing in 83.1% and decreasing in 16.9% of treatments. UF rates remained below 13 mL/kg/h in all simulations, and 99.4% of treatments remained within predefined UF volume limits. **Conclusions**: Adaptive UF improved intradialytic RBV target attainment and individualized UF delivery within predefined treatment constraints. Prospective studies are required to determine whether these physiological improvements translate into clinical benefit.

## 1. Introduction

Fluid overload remains one of the most critical and persistent challenges in the management of patients receiving maintenance hemodialysis. Chronic extracellular volume expansion contributes to hypertension, left ventricular hypertrophy, heart failure, hospitalization, and mortality, whereas excessive or poorly tolerated ultrafiltration (UF) may result in intradialytic hypotension, organ hypoperfusion, premature termination of treatment, and increased mortality risk [1,2,3,4,5]. Consequently, determining how much fluid should be removed at what rate remains one of the pivotal challenges of contemporary hemodialysis care [6,7].

In current U.S. clinical practice, the UF prescription is determined before treatment begins using information including interdialytic weight gain, estimated dry weight, previous treatment tolerance, cardiovascular status, and clinical assessment. The prescribed UF volume is then typically delivered using a constant or only modestly varying UF rate throughout the dialysis session [8]. This approach fundamentally assumes that a treatment prescription established before dialysis remains appropriate despite continuously changing patient physiology. In reality, intravascular volume status, plasma refill capacity, and cardiovascular responses evolve throughout treatment, with substantial variability both between patients and between repeated treatments in the same patient [9]. As a result, a UF prescription that appears appropriate at the start of dialysis may become suboptimal as a patient’s physiological responses change and adapt during the course of treatment. This mismatch raises a fundamental question: should ultrafiltration remain static throughout dialysis, or should it continuously adapt to the patient’s evolving physiological response?

Relative blood volume (RBV) monitoring provides a non-invasive method for continuously assessing changes in intravascular volume during dialysis. Numerous observational studies have demonstrated associations between RBV trajectories and plasma refill capacity, hemodynamic stability, hospitalization, cardiovascular outcomes, and mortality [2,10]. Despite its availability on many contemporary dialysis platforms, RBV monitoring is used in only a minority of U.S. treatments, in part because translating continuously changing physiological information into timely ultrafiltration adjustments requires sustained clinical attention and experience throughout the dialysis session. Although physiology-guided ultrafiltration strategies have been implemented outside the United States [11,12,13,14,15,16], routine U.S. fluid management remains largely based on treatment prescriptions established before dialysis begins, with ultrafiltration typically modified only after clinically significant events occur. Adaptive UF is a physiological closed-loop control strategy that addresses this limitation by continuously adjusting ultrafiltration according to the patient’s observed RBV trajectory while operating within clinician-defined treatment constraints. Rather than using RBV as a passive monitoring modality, Adaptive UF incorporates continuously acquired physiological information into real-time ultrafiltration management with the objective of maintaining RBV within a predefined target region associated with favorable clinical outcomes [17,18]. In this way, blood volume monitoring transitions from passive observation to active treatment support while preserving physician oversight through predefined treatment limits.

Because Adaptive UF remains an investigational technology, prospective clinical evaluation is not yet feasible. Patient-specific in silico methodologies provide a complementary approach by enabling novel treatment strategies to be evaluated under controlled conditions while preserving treatment-specific physiological characteristics derived from historical dialysis sessions. The objective of the present study was to evaluate Adaptive UF within a large patient-specific in silico framework derived from historical U.S. hemodialysis treatments. Specifically, we investigated whether continuous adaptation of ultrafiltration according to the observed RBV response could improve intradialytic RBV target attainment while individualizing fluid removal within clinician-defined treatment constraints.

## 2. Materials and Methods

### 2.1. Study Design and Data Source

**Figure 1:**
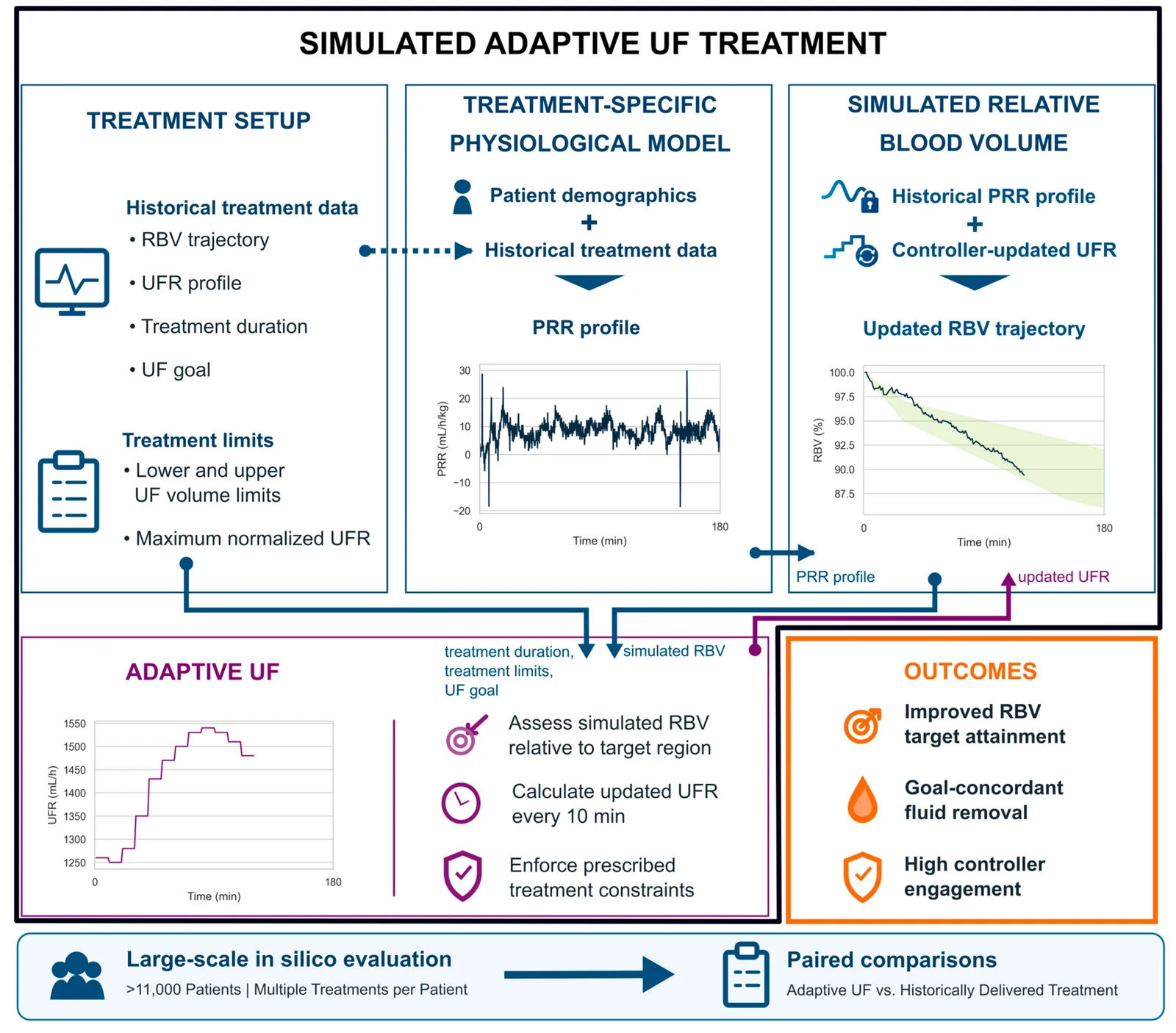
Simulated Adaptive UF treatment framework. Historical treatment data and treatment limits were used to reconstruct a treatment-specific plasma refill rate (PRR) profile and initialize the Adaptive UF simulation. The historical PRR profile was kept while the ultrafiltration rate (UFR) was updated by the controller every 10 min in response to simulated relative blood volume (RBV), generating an updated RBV trajectory. Outcomes included RBV target attainment, UF delivery, and controller engagement and limit adherence, with paired comparison against the corresponding historically delivered treatment. RBV, relative blood volume; PRR, plasma refill rate; UF, ultrafiltration; UFR, ultrafiltration rate. The figure was conceptualized by the authors and created in Inkscape 0.92.3.

This study used a paired patient-specific in silico framework to evaluate the potential impact of Adaptive UF compared with historically delivered hemodialysis treatments (Figure 1). Historical treatment data were obtained from a large U.S. dialysis database containing machine-recorded treatment information, including continuous RBV recordings measured with Crit-Line^®^ (Fresenius Medical Care North America, Waltham, MA, USA), from patients treated between January 2022 and April 2023.

Patients eligible for inclusion had at least 12 consecutive historical treatments available within less than 13 weeks. Treatment-level quality-control procedures excluded sessions outside the intended operating range of Adaptive UF, with prolonged missing data, or poor signal quality. Patients with fewer than six evaluable treatments after quality control were excluded. The study selection process is summarized in Supplementary Figure S1.

Each historically delivered treatment was analyzed as recorded and served as the clinical comparator. A corresponding simulated Adaptive UF treatment was generated using identical treatment characteristics within a patient-specific in silico framework, allowing direct paired comparison between the historically delivered and simulated treatments.

### 2.2. Treatment-Specific Physiological Reconstruction

For each historical treatment, machine-recorded ultrafiltration delivery, treatment duration, body weight, blood pressure measurements, and continuous RBV recordings were extracted and aligned to a uniform 10 s time grid. Missing observations were interpolated using predefined signal-specific procedures, whereas treatments failing quality-control criteria were excluded. Detailed preprocessing procedures are provided in the Supplementary Methods.

A treatment-specific physiological model was constructed by estimating the plasma refill rate (PRR) profile from the observed RBV trajectory together with the delivered ultrafiltration profile using previously described methods [19]; additional details are provided in the Supplementary Methods. The reconstructed PRR profile represents the patient’s physiological response during the historical treatment and served as the physiological model for the corresponding simulated Adaptive UF treatment. Thus, each simulated Adaptive UF treatment differed from the historically delivered treatment only in the method used to determine ultrafiltration delivery, allowing the isolated effect of Adaptive UF to be evaluated under otherwise identical treatment conditions.

### 2.3. Adaptive UF Simulation

Adaptive UF is a previously described physiological closed-loop control (PCLC) system that adjusts ultrafiltration delivery in real-time according to the patient’s observed RBV trajectory while operating within clinician-defined treatment constraints [18].

Each simulation was initialized using the corresponding historical treatment, including treatment duration, removed UF volume, and patient-specific estimated blood volume. The reconstructed plasma refill profile remained fixed throughout each simulated treatment.

Adaptive UF allows physicians to define lower and upper limits for the achieved ultrafiltration volume within a range extending up to 1000 mL below and 1000 mL above the prescribed ultrafiltration goal. Because these limits were not available in the historical dataset, treatment-specific limits were assigned for each simulation. Upper and lower limits were independently sampled as offsets from the prescribed ultrafiltration goal. To approximate routine clinical prescribing practice, where ultrafiltration limits are typically specified in 500 mL increments, 90% of sampled offsets were drawn from a coarse set of allowable values (500 and 1000 mL), whereas the remaining 10% were drawn from a finer set (100–1000 mL in 10 mL increments).

At each 10 min control interval, the controller received the current simulated RBV, elapsed treatment time, and cumulative ultrafiltration volume, and physician-defined treatment constraints. Based on these inputs, an updated ultrafiltration rate was calculated and applied to the treatment-specific physiological model, generating the corresponding simulated RBV response. This updated RBV was returned to the controller at the subsequent control interval, thereby forming a closed feedback loop throughout the simulated treatment.

The control objective was to maintain RBV within a predefined target region derived from previously reported associations between RBV trajectories and improved patient survival [17], while simultaneously respecting physician-defined ultrafiltration limits. In addition, Adaptive UF incorporates predefined safety features, including maximum normalized ultrafiltration rate, controller disengagement criteria, and blood pressure-based safety alerts. Blood pressure measurements were used exclusively within the safety logic and did not directly influence ultrafiltration control.

Further details regarding controller implementation are provided in the Supplementary Methods and previous publications [18,20].

### 2.4. Outcome Measures

The study evaluated whether Adaptive UF improved intradialytic RBV control while individualizing ultrafiltration delivery within predefined treatment constraints. Comparative outcome measures were initially calculated for each treatment and subsequently aggregated within patients for the primary analyses. Operational characteristics describing Adaptive UF behavior were summarized descriptively at the treatment level.

#### 2.4.1. Relative Blood Volume Target Attainment

The principal measure of RBV control was the percentage of treatment time during which RBV remained within the predefined favorable RBV target region derived from previously reported associations with improved patient survival [17]. RBV target attainment was evaluated at one-minute intervals using the RBV value at each minute boundary during an analysis window of 0–180 min.

Additional measures included the cumulative area and signed area outside target, reflecting the magnitude and direction of RBV excursions.

#### 2.4.2. Ultrafiltration Delivery

Measures of ultrafiltration delivery included achieved ultrafiltration volume and body weight-normalized ultrafiltration rate. Together with RBV target attainment, these outcomes were used to characterize how Adaptive UF individualized fluid removal.

#### 2.4.3. Adaptive UF Operational Characteristics

Operational characteristics included Adaptive UF engagement time, adherence to predefined ultrafiltration volume limits, and the magnitude of ultrafiltration limit exceedances. These measures were summarized descriptively at the treatment level.

### 2.5. Statistical Analysis

Because each historical treatment served as its own comparator, treatment effects were calculated as paired differences between the simulated Adaptive UF treatment and the corresponding historically delivered treatment. Comparative outcome measures were initially calculated for each treatment and subsequently averaged within each patient to obtain patient-level summaries representing the average treatment effect across the observation period. Patient-level aggregation ensured that each patient contributed equally to the primary analyses and prevented patients with larger numbers of evaluable treatments from disproportionately influencing the overall treatment effect.

Continuous variables are presented as mean ± standard deviation (SD), or as median (interquartile range [IQR]) for skewed distributions, and categorical variables as counts and percentages. Mean paired differences are reported together with corresponding 95% confidence intervals. Patient-level differences between Adaptive UF and historically delivered treatments were evaluated using paired *t*-tests, with Wilcoxon signed-rank tests being performed as a non-parametric sensitivity analysis to assess the robustness of the findings. As an additional sensitivity analysis, patient-level differences were also evaluated using a treatment-level linear mixed-effects model with a patient-level random intercept to account for repeated treatments within patients to assess consistency; details are provided in the Supplementary Material.

Because the study included a large number of patients (>11,000) and treatments (>119,000), statistical significance was interpreted conservatively, as even small numerical differences may result in very small P values in this study.

The baseline for the study population characterization was defined as the time of the first included treatment per patient. History of comorbidities is defined as recorded diagnosis before or at baseline.

Consistency and heterogeneity of treatment effects were visualized using waterfall plots together with patient- and treatment-level distribution plots. Operational characteristics of Adaptive UF were summarized descriptively at the treatment level and were not included in comparative statistical analyses.

All simulated treatments were analyzed over the full prescribed treatment duration regardless of controller disengagement. In the case of disengagement, the simulation continued using the ultrafiltration rate last generated by Adaptive UF immediately before disengagement; see Supplemental Methods for further detail.

All data processing and statistical analyses were performed using Python 3.13.5 with NumPy 2.2.6, pandas 2.3.0, and statsmodels 0.14.6. Visualizations were generated using Matplotlib 3.10.3 and Seaborn 0.13.2.

## 3. Results

### 3.1. Study Population

The study population comprised 11,246 patients contributing 119,472 hemodialysis treatments following application of predefined inclusion criteria and quality-control criteria. The treatments included in the analysis were derived from routine maintenance hemodialysis care in which dialysis adequacy is monitored monthly according to standard clinical practice. On average, each patient contributed 10.6 ± 1.7 treatments.

Patient and treatment characteristics are summarized in Table 1. The study population encompassed a broad range of body weights, treatment durations, ultrafiltration prescriptions, and blood pressure characteristics representative of routine U.S. in-center hemodialysis practice.

### 3.2. Adaptive UF Improves Relative Blood Volume Target Attainment

**Figure 2:**
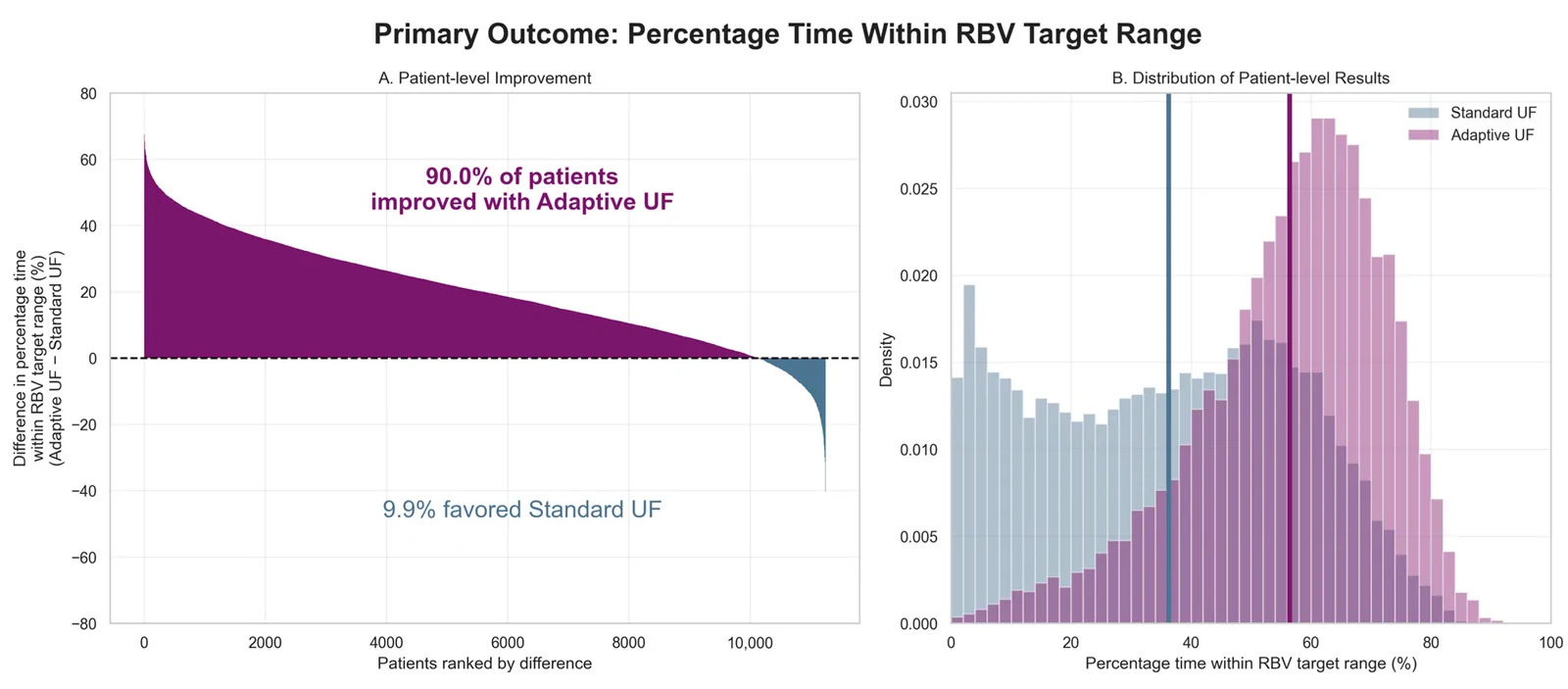
Adaptive UF improves relative blood volume (RBV) target attainment. (**A**) Patient-level mean differences in RBV target attainment (Adaptive UF minus historically delivered treatment), ordered from greatest improvement to greatest reduction. (**B**) Distribution of treatment-level differences in RBV target attainment. Positive values indicate greater RBV target attainment with Adaptive UF.

Adaptive UF substantially increased RBV target attainment compared with historically delivered treatments. Mean time within the predefined favorable RBV target region increased from 36.3% during historically delivered treatments to 56.4% during simulated Adaptive UF treatments, corresponding to an absolute improvement of 20.2 percentage points (95% CI, 19.9–20.5) and a relative improvement of 55.7%. RBV target attainment increased in 90.0% of patients, indicating that the improvement was broadly distributed across the study population (Figure 2). Both paired *t*-tests and Wilcoxon signed-rank tests yielded *p* < 0.001 (Table 2).

These improvements were accompanied by reductions in both the magnitude and duration of RBV excursions outside the target region. The mean cumulative area outside the target range decreased from 280.7 to 139.5 RBV %–minutes, corresponding to a mean paired reduction of 141.3 RBV %–minutes. Area outside the target region decreased in 90.5% of patients.

Patient-level RBV-related outcomes are summarized in Table 2. Results were consistent in the linear mixed-effects sensitivity analysis (Supplementary Table S4).

### 3.3. Adaptive UF Individualizes Ultrafiltration Delivery

Improvements in RBV target attainment were accompanied by changes in ultrafiltration delivery. Mean achieved UF volume increased from 2620.7 mL during historically delivered treatments to 2917.1 mL with Adaptive UF, corresponding to a mean paired increase of 296.4 mL (Table 2).

**Figure 3:**
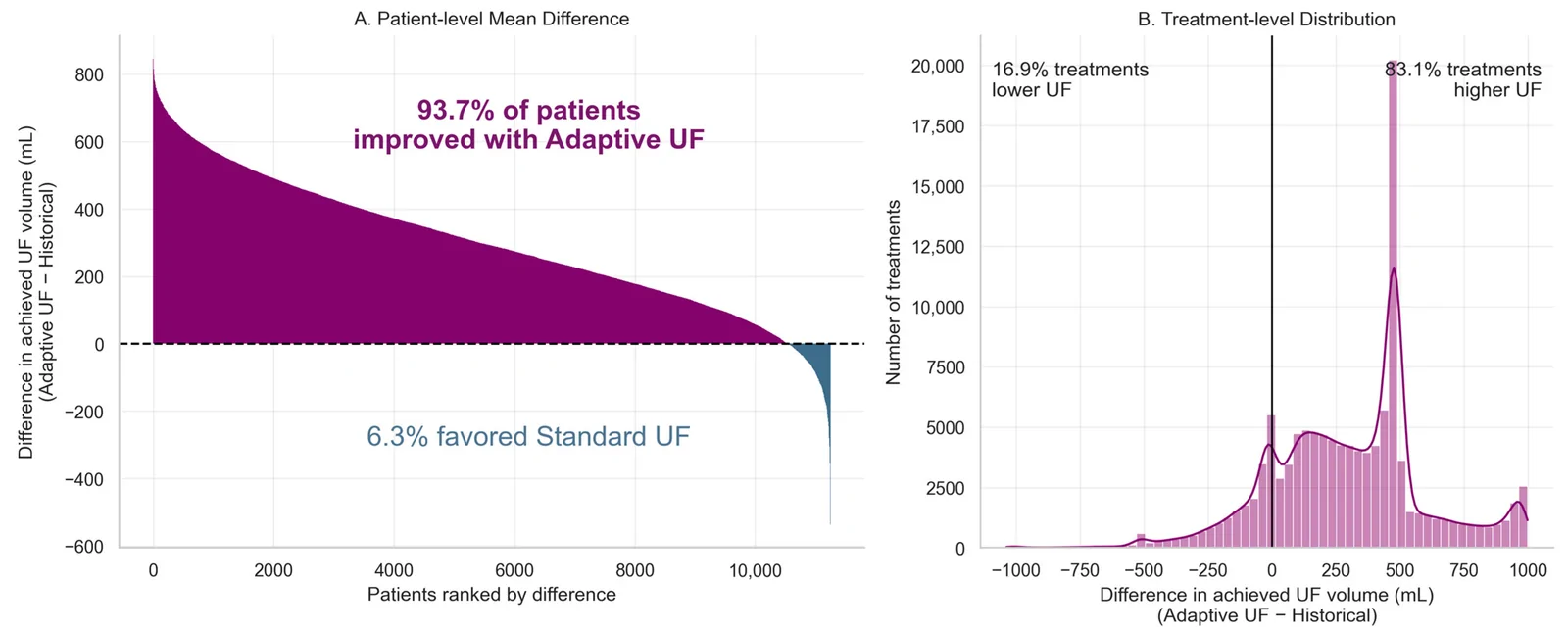
Adaptive UF individualizes ultrafiltration delivery. (**A**) Patient-level mean difference in achieved ultrafiltration volume (Adaptive UF minus historically delivered treatment), ordered from largest increase to largest decrease. (**B**) Distribution of treatment differences in achieved ultrafiltration volume. Positive values indicate greater ultrafiltration volume with Adaptive UF; negative values indicate lower ultrafiltration volume.

The increase in UF volume was not uniform across treatments. At the treatment level, achieved UF volume increased relative to the corresponding historical treatment in 83.1% of treatments and decreased in 16.9% (Figure 3B). After patient-level aggregation, mean UF volume was higher with Adaptive UF in 93.7% of patients and lower in 6.3% (Figure 3A). Thus, despite an overall increase in fluid removal, Adaptive UF modified UF delivery in both directions across individual treatments. The broad distribution of treatment effects illustrates substantial heterogeneity in ultrafiltration requirements both between patients and across repeated treatments within individual patients, as would be expected.

Mean body weight-normalized UF rate increased from 8.5 to 9.4 mL/kg/h. UF rates remained below the predefined maximum of 13 mL/kg/h in all simulated treatments.

Patient-level ultrafiltration outcomes are summarized in Table 2. Results were consistent in the linear mixed-effects sensitivity analysis (Supplementary Table S4).

### 3.4. Adaptive UF Operational Characteristics

**Figure 4:**
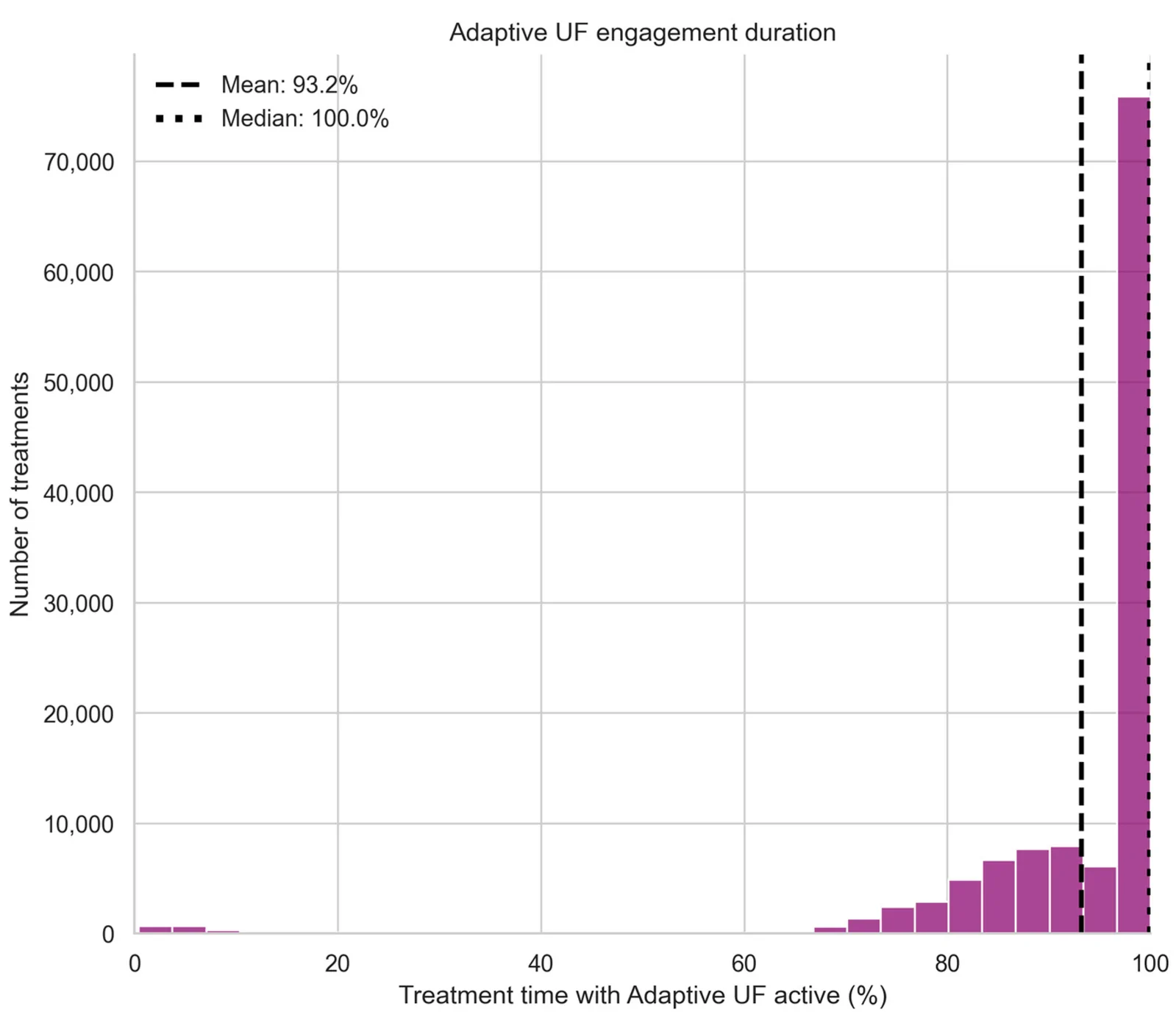
Adaptive UF engagement during simulated treatments. Distribution of the percentage of treatment time during which Adaptive UF remained engaged. Dashed and dotted vertical lines indicate the mean and median, respectively.

Adaptive UF remained engaged for a mean of 93.2% of treatment duration (median 100%, IQR 90.2–100.0%), with physiological feedback control therefore active throughout most simulated treatments (Figure 4). By design, Adaptive UF disengages when predefined safety criteria were met, as detailed in the Supplementary Methods.

Achieved ultrafiltration volume remained within the predefined UF volume limits in 99.4% of treatments. Among treatments ending outside these limits, the mean absolute exceedance was 14.0 ± 7.5 mL. Treatment-level controller performance metrics are summarized in Table 3.

## 4. Discussion

### 4.1. Principal Findings

In this large patient-specific in silico study, Adaptive UF substantially improved intradialytic RBV target attainment compared with historically delivered hemodialysis treatments while individualizing ultrafiltration delivery within predefined treatment constraints. Patients spent more treatment time within the favorable RBV target region, and the cumulative burden of excursions outside the target region was markedly reduced. These effects were observed across the majority of patients rather than being driven by a small subset of responders.

Improved RBV control was accompanied by bidirectional changes in ultrafiltration delivery. Although achieved ultrafiltration volume increased on average, Adaptive UF both increased and decreased fluid removal across individual treatments according to the observed RBV response. Thus, the principal finding is not simply that Adaptive UF altered the amount of fluid removed, but that physiological feedback allowed ultrafiltration delivery to adapt dynamically during treatment.

Together, these findings support the feasibility of using continuously acquired physiological information to individualize ultrafiltration delivery rather than relying exclusively on a treatment prescription established before dialysis begins.

### 4.2. Moving Beyond Static Ultrafiltration Prescriptions

Conventional ultrafiltration prescribing in U.S. hemodialysis practice is largely based on a treatment plan established before dialysis, whereas the physiological conditions governing fluid removal evolve throughout the session. Plasma refill capacity, intravascular volume, and cardiovascular responses may change substantially during treatment and between repeated treatments in the same patient. A fixed prescription therefore cannot account directly for this evolving physiological response.

The bidirectional behavior observed in the present study illustrates the potential consequence of introducing physiological feedback into this process. Adaptive UF did not systematically increase or decrease fluid removal; instead, ultrafiltration delivery changed according to the evolving RBV trajectory. Although independent measures of fluid status were unavailable, this behavior is consistent with substantial heterogeneity in fluid removal requirements across patients and treatments. Chronic fluid overload is common among U.S. hemodialysis patients, whereas a much smaller proportion appear volume depleted before treatment [21]. Within this heterogeneous population, the ability to adapt fluid removal to treatment-specific physiology may offer advantages over relying exclusively on a predetermined UF prescription.

More broadly, Adaptive UF represents one implementation of a physiology-guided treatment paradigm in which continuously acquired physiological information is incorporated into dialysis therapy in real time. Automated blood-volume-guided ultrafiltration systems have previously been implemented outside the U.S., providing clinical precedent for incorporating physiological feedback into UF delivery [22,23,24]. The relevance of the present findings extends beyond a specific control algorithm and supports further investigation of physiological feedback as a strategy for individualizing fluid management during hemodialysis.

### 4.3. Clinical Implications

The present findings are particularly relevant in the context of contemporary U.S. hemodialysis practice, where chronic fluid overload remains common despite widespread use of standardized ultrafiltration prescriptions. The U.S. represents one of the world’s largest hemodialysis populations and continues to experience a substantial burden of fluid-related complications. At the same time, chronic fluid overload remains highly prevalent, highlighting the need for approaches that can individualize fluid management beyond a single ultrafiltration prescription established before treatment begins.

Observational studies have demonstrated differences between overall RBV trajectories observed during treatment and clinically significant outcomes, including intradialytic hypotension [25]. Blood volume-guided ultrafiltration systems have been implemented outside the United States, demonstrating the feasibility of incorporating physiological feedback into UF delivery [11,12,13,14,15,16], and reductions in intradialytic hypotensive events have been reported with blood-volume-guided treatment strategies [22,23,24,26]. Nevertheless, RBV monitoring is not routinely incorporated into ultrafiltration management in U.S. clinical practice, where translating continuously changing physiological information into treatment adjustments requires clinical interpretation and sustained attention throughout dialysis.

Adaptive UF extends blood volume monitoring from passive observation to active physiological guidance by incorporating RBV measurements into ultrafiltration management while operating within physician-defined treatment limits. Importantly, such an approach is intended to augment rather than replace clinical decision-making: the treatment prescription and allowable boundaries remain clinically established, while physiological feedback determines how fluid removal is adjusted within those boundaries.

The present study does not aim to establish clinical benefit. However, the improvement in RBV target attainment across a large and heterogeneous U.S. hemodialysis population suggests that physiology-guided ultrafiltration can meaningfully alter intradialytic blood volume control. Whether these physiological improvements translate into better treatment tolerance, fluid management, or clinical outcomes requires prospective evaluation.

### 4.4. Strengths and Limitations

A principal strength of this study is the paired design, in which each simulated Adaptive UF treatment was compared directly with the corresponding historically delivered treatment under otherwise identical treatment conditions. This within-treatment comparison minimized variability arising from differences in patient characteristics, treatment prescriptions, and underlying physiology. The large U.S. cohort of more than 11,000 patients and nearly 120,000 treatments further enabled evaluation across a heterogeneous hemodialysis population. Finally, the patient-specific in silico framework incorporated treatment-specific physiological models reconstructed from the corresponding historical treatment, preserving observed plasma refill characteristics across individual dialysis sessions.

Several limitations should be considered. Most importantly, this was an in silico study and cannot reproduce all aspects of clinical treatment. The reconstructed plasma refill profile was assumed to remain unchanged during each simulated treatment; consequently, the model did not account for potential changes in refill characteristics resulting from the altered UF delivery itself. Independent measures of fluid status (e.g., bioimpedance) were unavailable, preventing assessment of whether treatments with increased or decreased UF corresponded to differences in underlying volume status. In addition, the RBV target region was derived from observational associations and requires prospective validation. Finally, clinical outcomes such as intradialytic symptoms, hospitalization, and mortality were not evaluated, and no conclusions regarding clinical benefit can be drawn from these simulations.

### 4.5. Future Directions

The present findings provide a rationale for prospective clinical evaluation of physiology-guided ultrafiltration. Future studies should determine whether improved RBV target attainment translates into improvements in treatment tolerance, intradialytic symptoms and hemodynamics, fluid management, and patient outcomes. More broadly, prospective investigation will be necessary to establish whether physiological feedback can be safely and effectively integrated into routine hemodialysis and to define its role within precision fluid management.

## 5. Conclusions

Adaptive UF substantially improved intradialytic RBV target attainment while individualizing ultrafiltration delivery within predefined treatment constraints in a large patient-specific in silico framework derived from U.S. hemodialysis treatments. The bidirectional adaptation of fluid removal illustrates the potential of physiological feedback to move ultrafiltration management beyond static treatment prescription towards therapy responsive to evolving patient physiology. Prospective studies are required to determine whether these physiological improvements translate into clinical benefit.

## 6. Patents

The Adaptive UF technology evaluated in this study is related to U.S. Patent No. 12,594,366, Techniques for Dialysis Based on Relative Blood Volume, issued 7 April 2026, to inventors Doris H. Fuertinger, Peter Kotanko, and Sabrina Rogg (now Sabrina Casper), and assigned to Fresenius Medical Care Holdings, Inc. and Fresenius Medical Care Deutschland GmbH.

## Figures and Tables

**Table 1:** Patient and treatment characteristics.

Characteristic	Study Population (N = 11,246)
Number of treatments per patient	10.6 ± 1.7
Female sex, n (%)	4312 (38.3)
Weight ^a^, kg	84.8 ± 20.4
Height ^a^, cm	168.8 ± 13.9
Treatment duration, min	223.1 ± 16.9
UF volume, mL	2620.7 ± 740.4
UFR, mL/kg/h	8.5 ± 1.9
Pre-dialysis SBP, mmHg	141.6 ± 21.0
Intradialytic min SBP, mmHg	108.4 ± 20.3
Intradialytic max SBP, mmHg	157.4 ± 20.6
Post-dialysis SBP, mmHg	131.1 ± 19.7
Estimated initial blood volume, mL	6049.1 ± 1555.8
Mean interdialytic weight gain (IDWG) ^b^, kg	2.4 ± 0.8
Dialysis vintage ^a,c^, days	1069 (436, 2059)
HGB ^a^, g/dL	10.9 ± 1.2
History of diabetes ^a^, n (%)	7612 (67.7)
History of hypertension ^a^, n (%)	9536 (84.8)
History of COPD ^a^, n (%)	1150 (10.2)
Access type used	
Central venous catheter (CVC) ^a^, n (%)	2721 (24.2)
Other access types (arteriovenous fistula	8525 (75.8)
[AVF] or arteriovenous graft [AVG]) ^a^, n (%)	

^a^ at baseline; ^b^ patient-level mean; ^c^ reported as median (IQR).

**Table 2:** Patient-level comparative analysis.

Outcome	Historically Delivered Treatment	Adaptive UF	Mean Difference (95% CI)	*t*-Test *p*-Value	Wilcoxon *p*-Value
Number of treatments per patient	10.6 ± 1.7	10.6 ± 1.7	NA	NA	NA
**RBV target attainment**					
Time within RBV target range, %	36.3 ± 21.3	56.4 ± 15.7	20.2 (19.9, 20.5)	<0.001	<0.001
Area outside target range, RBV %–minutes	280.7 ± 224.1	139.5 ± 136.3	−141.3 (−144.1, −138.5)	<0.001	<0.001
Signed area outside target range, RBV %–minutes	155.6 ± 281.0	52.8 ± 153.6	−102.8 (−106.3, −99.4)	<0.001	<0.001
**Ultrafiltration Delivery**					
Achieved UF volume, mL	2620.7 ± 740.4	2917.1 ± 761.4	296.4 (292.8, 300.1)	<0.001	<0.001
UF rate, mL/kg/h	8.49 ± 1.94	9.41 ± 1.80	0.9 (0.9, 0.9)	<0.001	<0.001

**Table 3:** Operational characteristics of Adaptive UF (treatment-level analysis).

Operational Characteristic	Adaptive UF
Number of treatments	119,472
Adaptive UF engagement, % treatment time	93.2 ± 14.5
Treatments within predefined UF volume limits, n (%)	118,754 (99.4%)
Absolute UF volume limit exceedance *, mL	14.0 ± 7.5
Minimum limit exceedance	0.03

* Calculated among treatments that exceeded prescribed UF limits.

## Data Availability

The data that support the findings of this study may be available upon reasonable request and subject to applicable data-use and confidentiality restrictions.

## Supplementary Materials

The following supporting information can be downloaded at https://www.mdpi.com/article/10.3390/jcm15187078/s1: Supplementary Methods; Figure S1: Exclusion flow diagram for cohort construction; Figure S2: Treatments per patient; Figure S3: Missingness among affected treatments; Figure S4: Duration of missing segments; Figure S5: Favorable RBV target range; Table S1: Missingness for RBV imputation; Table S2: Missingness for UFR imputation; Table S3: Overview of simulation parameters and settings; Table S4: Outcomes of the linear mixed-model analyses. References [27,28] are cited in the supplementary materials.

## Abbreviations

The following abbreviations are used in this manuscript:

PCLC	physiological closed-loop control
PRR	plasma refill rate
RBV	relative blood volume
UF	ultrafiltration
UFR	ultrafiltration rate
U.S.	United States of America
