# Supplementary material for: Toward Precision Fluid Management in Hemodialysis: Adaptive Ultrafiltration Guided by Continuous Relative Blood Volume Monitoring
Source: J Clin Med. 2026 Sep 12;15(18):7078. doi: 10.3390/jcm15187078 (PMC13607670; doi:10.3390/jcm15187078)
Supplement: Supplementary file 1 [file jcm-15-07078-s001.zip › jcm-4533922-supplementary.pdf]

SUPPLEMENTARY MATERIAL

Table of Contents

Supplementary Methods: 1
Cohort construction and treatment-level quality control 1
Alignment and imputation of machine-recorded RBV and UF rate data 3
Plasma refill rate reconstruction 5
Treatment end correction........ 5
Adaptive UF controller implementation in the simulation framework 6
Favorable RBV target range 7
Supplementary analysis: 8
Treatment-level linear mixed model........ **Error! Bookmark not defined.**
Discussion........ **Error! Bookmark not defined.**

Supplementary Methods

Comment: Agreeing with the style guide of the journal of clinical medicine, all references are numbered in agreement with the reference list in the main manuscript.

Cohort construction and treatment-level quality control

The source dataset included 13,214 patients with at least 12 recorded hemodialysis treatments within a period of less than 13 weeks, corresponding to 158,568 treatments selected for analysis. Treatment-level quality-control criteria were then applied sequentially. Treatments were excluded if treatment duration was less than 180 minutes or greater than 270 minutes, if average ultrafiltration rate was ≤300 mL/h, if average ultrafiltration rate was ≥13 mL/kg/h, if post-treatment weight was less than 40 kg or greater than 160 kg, if missing data gaps of 20 minutes or longer were present, or if three or more infusions were suspected. Treatments also underwent preprocessing for interpolation of missing data and application of a treatment end correction (see Supplementary Method Section “Treatment end correction”) required for generation of treatment-specific in-silico refill profiles.

After treatment-level quality control, 124,470 treatments from 12,831 patients remained. Patients with fewer than six analyzable treatments were excluded from the primary analysis. This excluded 4,998 treatments from 1,585 patients, resulting in a final analysis population of 119,472 treatments from 11,246 patients.

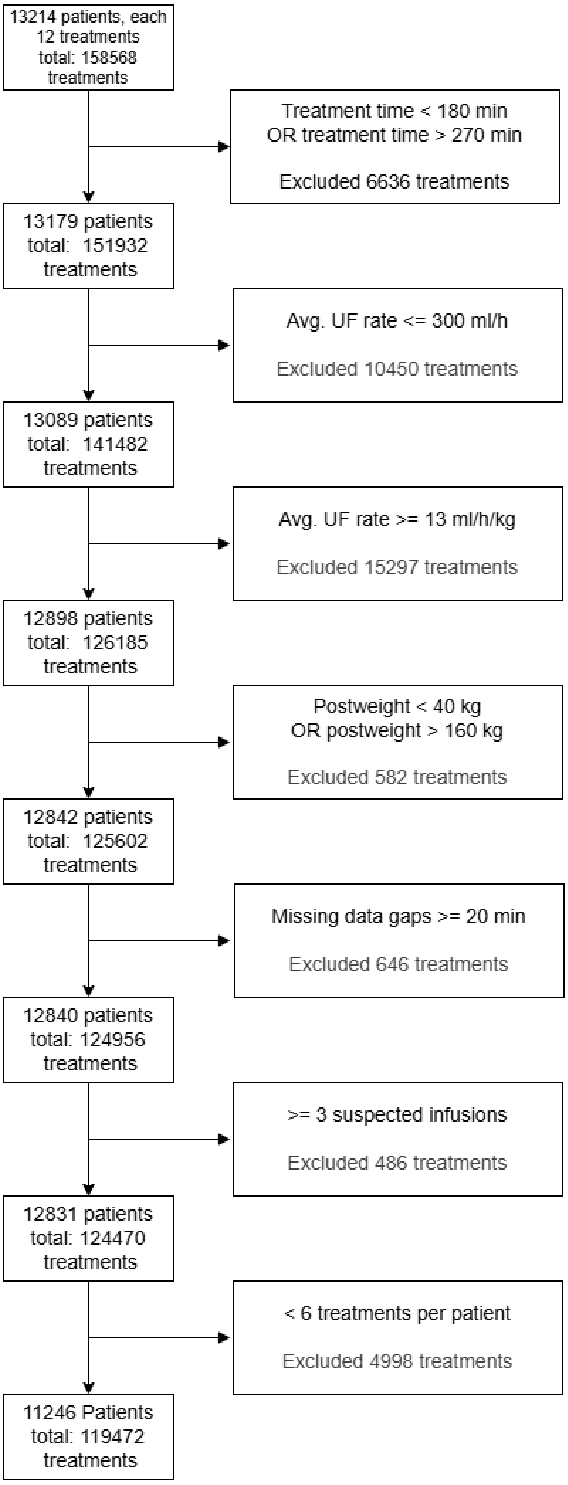

Supplementary Figure S1. Exclusion flow diagram for cohort construction.
Treatments were excluded for implausible treatment duration, ultrafiltration rate, post-treatment weight, prolonged missing data gaps, or excessive abrupt RBV changes. Patients with fewer than six analyzable treatments after quality control were excluded. The final analysis population included 11,246 patients and 119,472 treatments.

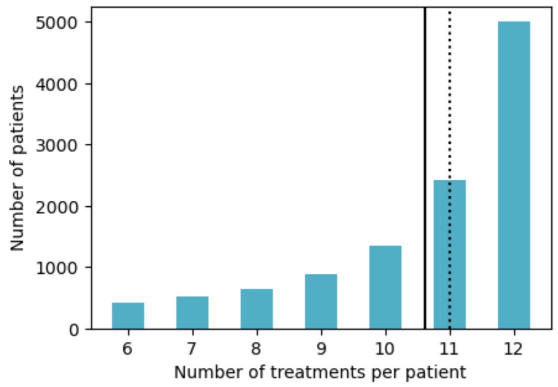

Supplementary Figure S2. Treatments per patient
Distribution of the number of treatments per patient displayed as histogram. The solid line indicates the mean, and the dotted line indicates the median.

Alignment and imputation of machine-recorded RBV and UF rate data

Machine-recorded RBV and UF rate data were available at approximately 10-second resolution. For each treatment, recorded time stamps were mapped to a fixed 10-second time grid beginning at treatment start. Time points without a recorded RBV or UF rate value were explicitly represented as missing values before imputation. Machine-recorded time stamps were unique within each treatment. If recording started after treatment initiation, the initial RBV value was set by definition to 0% reduction from baseline, and missing UF rate values before the first available UF rate measurement were imputed using the earliest available UF rate value.

Missing RBV values were imputed using local linear interpolation. Consecutive missing RBV segments were identified, and for each missing segment the mean RBV value in the preceding 5-minute window and the mean RBV value in the following 5-minute window were calculated, where available. If valid values were available on both sides of the missing segment, missing RBV values were linearly interpolated between these two local mean values. Missing RBV segments without valid data on both sides were not imputed by this procedure.

Missing UF rate values were imputed using a stepwise approach reflecting the piecewise-constant nature of machine-delivered UF rate settings. For missing UF rate segments occurring after at least one valid UF rate value, the most recent preceding UF rate value was carried forward across the missing segment. If missing UF rate values occurred at the start of treatment, the first subsequent valid UF rate value was carried backward. This approach preserved abrupt UF rate changes when recorded while avoiding artificial smoothing of the UF delivery profile.

No denoising or smoothing was applied to the RBV trajectory beyond the described missing-data interpolation. Treatments with prolonged missing data gaps of 20 minutes or longer were excluded during treatment-level quality control (See supplementary methods section on cohort construction and treatment-level quality control).

Supplementary Table S1. Missingness for RBV imputation.
Summary of missing relative blood volume (RBV) data before imputation among included treatments, including the proportion of treatments with missing values, overall and treatment-level missingness, and the duration of consecutive missing RBV segments.

Measure	Value
Treatments evaluated, n	119,472
Treatments with any missing RBV values, n (%)	113,298 (94.8%)
Missing RBV time points overall, %	2.48%
Missing RBV time points per treatment, median (IQR), %	0.96% (0.38–2.34%)
Missing-RBV-segment duration, median (IQR), min	0.33 (0.17–0.83)
Missing-RBV -segment duration, 95th percentile, min	1.67

Supplementary Table S2. Missingness for UFR imputation.
Summary of missing ultrafiltration (UF) rate data before imputation among included treatments, including the proportion of treatments with missing values, overall and treatment-level missingness, and the duration of consecutive missing UF-rate segments.

Measure	Value
Treatments evaluated, n	119,472
Treatments with any missing UF-rate values, n (%)	83,981 (70.3%)
Missing UF-rate time points overall, %	0.44%
Missing UF-rate time points per treatment, median (IQR), %	0.14% (0.00–0.33%)
Missing-segment duration, median (IQR), min	0.17 (0.17–0.17)
Missing-segment duration, 95th percentile, min	0.50

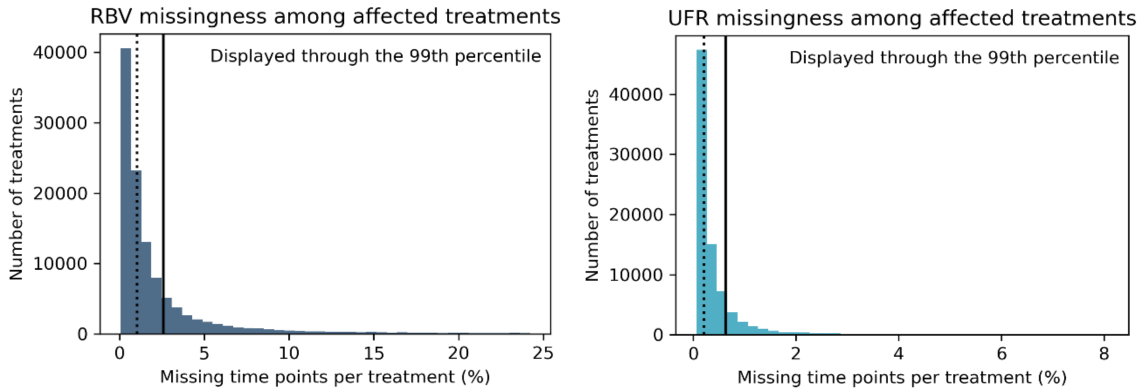

Supplementary Figure S3. Missingness among affected treatments.
Distribution of nonzero missing time points per treatment for **RBV (left)** and **UFR (right)**. Histograms show affected treatments only and are displayed through the 99th percentile. Solid and dotted vertical lines indicate the mean and median, respectively.

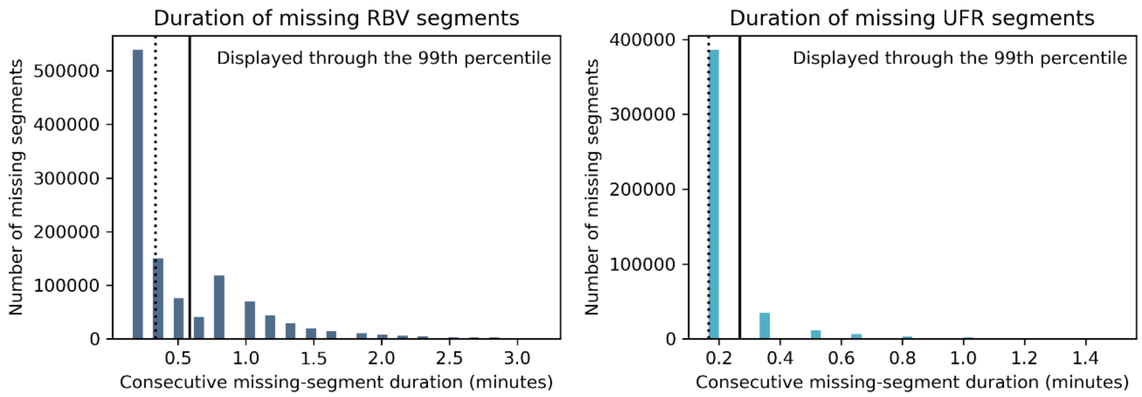

Supplementary Figure S4. Duration of Missing Segments.
Distribution of consecutive missing-segment durations for **RBV (left)** and **UFR (right)**. Histograms show missing-segment durations in minutes and are displayed through the 99th percentile. Solid and dotted vertical lines indicate the mean and median, respectively.

Reconstruction of treatment-specific plasma refill rates

Patient-level plasma refill rate profiles were reconstructed from the imputed RBV trajectory and delivered UF rate. Initial blood volume was estimated from post-treatment body weight and sex using a sex-specific weight-based approximation of 65 mL/kg for female patients and 75 mL/kg for male patients [27,28]. Machine-recorded RBV reduction was converted to absolute blood volume reduction by multiplying the percentage RBV reduction by the estimated initial blood volume. The time-dependent absolute blood volume reduction rate was then calculated from the first difference of absolute blood volume reduction on the 10-second grid and converted to mL/min. Plasma refill rate was estimated as the sum of the absolute blood volume reduction rate and the delivered UF rate, with UF rate expressed in mL/min.

No denoising or additional smoothing was applied to the RBV trajectory or reconstructed refill-rate profile beyond the prespecified missing-data interpolation.

During Adaptive UF simulation, the reconstructed plasma refill profile was assumed to remain unchanged from the historical treatment and therefore represented the treatment-specific physiological response against which alternative ultrafiltration profiles were evaluated.

Treatment end correction

Some treatments contained a terminal period in which UF rate was reduced to very low levels, consistent with an end-of-treatment phase rather than the intended UF delivery period. To avoid including this terminal phase in refill-rate reconstruction and outcome analysis, the end of the analyzable treatment was determined from the UF rate profile. Within a prespecified window at the end of treatment, the UF rate profile was searched backward for a transition from active UF delivery to a UF rate of ≤5 mL/min. If such a transition was detected after a period with UF rate >5 mL/min, the analyzable treatment was truncated at the last time point before the low-UF terminal phase. After truncation, the treatment end was aligned to the last full-minute time point.

Adaptive UF controller implementation in the simulation framework

Adaptive UF was implemented using a previously described proportional-integral feedback controller designed to guide the RBV trajectory toward a predefined favorable target region while respecting physician-defined treatment constraints [18]. The present study focused on evaluating controller performance within a patient-specific in silico framework rather than re-describing the control algorithm itself.

Each simulation was initialized using the corresponding historical treatment prescription, including treatment duration, prescribed ultrafiltration (UF) volume, and patient-specific estimated blood volume and hematocrit. The reconstructed plasma refill rate profile derived from the historical treatment was assumed to represent the patient's physiological response throughout the simulation and remained fixed during each simulated treatment.

In clinical use, Adaptive UF allows physicians to define lower and upper limits for the achieved UF volume within a range extending up to 1000 mL below and 1000 mL above the prescribed UF goal. These physician-defined limits constrain the range over which the controller may adjust fluid removal during treatment. For example, a prescribed UF goal of 3500 mL could be associated with allowable limits ranging from 2500 to 4500 mL.

Because physician-defined UF limits were not available in the historical dataset (these are inherent in clinical practice when estimating total treatment UF volume), treatment-specific limits were assigned for each simulation. Upper and lower limits were sampled independently as offsets from the prescribed UF goal. To approximate routine clinical prescribing practice, where UF limits are typically specified in coarser increments rather than finer adjustments, 90% of sampled offsets were drawn from a coarse set of allowable values (500 and 1000 mL), while the remaining 10% were drawn from a finer set (100, 110, 120, ..., 1000 mL). This approach introduced realistic variability in physician-defined treatment constraints while reflecting typical clinical use.

The controller continuously received the simulated RBV values, prescribed UF goal, elapsed treatment time, remaining treatment time, cumulative UF volume delivered, and the predefined treatment constraints. Based on these inputs, the controller calculated an updated UF-rate command every 10 minutes. This command was then applied to the patient-specific physiological model, which generated the subsequent RBV response using the reconstructed plasma refill profile. The updated RBV value was returned to the controller at the next control interval, thereby forming a closed feedback loop throughout the simulated treatment.

The controller operated within physician-defined treatment constraints on lower and upper UF volume limits, specified individually for each treatment, and a general weight-normalized UF rate limit. Depending on the simulated treatment state, the controller could increase, decrease, or maintain the current UF rate while ensuring that prescribed operating limits were not exceeded. When predefined safety criteria were met, the controller either temporarily suspended UF-rate updates, entered a predefined fallback mode, or disengaged. Following controller disengagement, the simulation continued using the last commanded UF rate for the remainder of the treatment.

For the in-silico implementation, disengagement criteria were limited to conditions that could be evaluated from variables represented in the simulation. Specifically, under predefined conditions, a scheduled controller update could be skipped, in which case no new UF-rate command was generated for that interval. If three consecutive controller updates were skipped,

Adaptive UF control was disengaged. Controller disengagement could also occur independently when predefined RBV safety criteria were met. Specifically, the controller was disengaged when simulated RBV remained ≤90% of baseline for at least 30 seconds before 1 hour of UF time had elapsed, or ≤85% of baseline for at least 30 seconds after 1 hour of UF time had elapsed. Following disengagement, no further controller-generated UF-rate updates were performed.

In the present in-silico implementation, the controller was not connected to real-time patient measurements. Instead, it acted on the simulated RBV trajectory generated from the reconstructed treatment-specific plasma refill profile and the controller-generated UF-rate profile.

Supplementary Table S3. Overview of simulation parameters and settings.

Parameter	Simulation setting
Controller type	Proportional–integral RBV feedback controller
UF-rate update interval	10 min
RBV input resolution	10 s
RBV target	Predefined favorable RBV region [17]
Maximum normalized UFR	13 mL/kg/h
aUF update-skip criterion 1	UF rate at minimum for ≥25 s during the final minute of the 10-min update interval
aUF update-skip criterion 2	UF rate at minimum for ≥2.5 min during the current 10-min update interval
aUF update-skip criterion 3	Hematocrit decrease >5.0 units within 2 min
aUF disengagement criterion 1 (skipped updates)	3 consecutive skipped controller updates
aUF disengagement criterion 2 (RBV, <1 h UF time)	RBV ≤90% of baseline for ≥30 s
aUF disengagement criterion 3 (RBV, ≥1 h UF time)	RBV ≤85% of baseline for ≥30 s
Prescribed UF goal	Achieved UF volume in historical treatment
Allowable UF-volume range	Treatment-specific lower and upper UF limits around prescribed UF goal
Plasma refill response	Reconstructed from historical RBV and UF
Weight	Historical treatment data
Treatment duration	Historical treatment data
Controller behavior after disengagement	Last controller-generated UF rate maintained for the remainder of the simulated treatment

Favorable RBV target range

The favorable RBV target range was based on previously reported half-hourly RBV ranges associated with lower all-cause mortality in maintenance hemodialysis patients. RBV was expressed as percentage of baseline blood volume, with 100% corresponding to treatment start. A continuous target region was created by linearly connecting the upper and lower half-hourly target bounds reported by Preciado et al. [17]. The primary endpoint was the percentage of treatment time during which the reconstructed or simulated RBV trajectory remained within this target region.

The target range was evaluated through 180 minutes using the following anchor points: 100% at treatment start, 95–97% at 30 minutes, 93–96% at 60 minutes, 91–95% at 90 minutes, 89–94% at 120 minutes, 87–93% at 150 minutes, and 86–92% at 180 minutes.

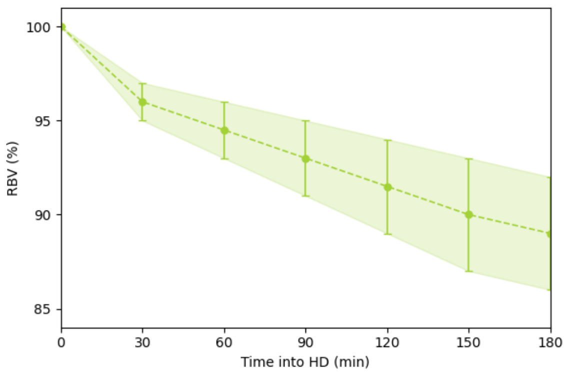

Supplementary Figure S5. Favorable RBV target range.
Illustration of the favorable RBV target range as reported by Preciado et al. [17].

Supplementary analysis

Treatment-level linear mixed-model analyses

Treatment-level paired differences were analyzed using linear mixed models to account for repeated treatments within patients. For each treatment, the paired difference was calculated as the outcome during Adaptive UF simulation minus the corresponding value during the historically delivered treatment. Separate models were fitted for percentage of treatment time within the RBV target range, area outside the RBV target range, signed area outside the RBV target range, achieved UF volume, and weight-normalized UF rate.

All models followed the same random-intercept model structure:

$$D_{ij} = \alpha_0 + u_i + \epsilon_{ij}$$

where D_{ij} denotes the paired treatment-level difference for treatment (j) in patient (i), α_0 is the mean paired treatment effect, u_i is a patient-specific random intercept, and ϵ_{ij} is the residual treatment-level error. The model was fitted using restricted maximum likelihood. The patient-level random intercept accounted for correlation among repeated treatment differences from the same patient.

A total of 119,472 treatments from 11,246 patients were included in each treatment-level mixed-model analysis. Adaptive UF was associated with an increase of 20.2 percentage points in time within the RBV target range (95% CI, 19.9 to 20.5; $p<0.001$). Area outside the RBV target range was reduced by 141.1 units (95% CI, −143.9 to −138.2; $p<0.001$), and signed area outside the target range was reduced by 102.7 units (95% CI, −106.1 to −99.3; $p<0.001$). Achieved UF volume was 297.2 mL greater with Adaptive UF (95% CI, 293.5 to 300.8; $p<0.001$), and weight-normalized UF rate was 0.93 mL/kg/h greater (95% CI, 0.91 to 0.94; $p<0.001$). These treatment-level estimates were consistent with the corresponding patient-aggregated comparisons.

Supplementary Table S4. Outcomes of the linear mixed-model analyses.
Treatment-level linear mixed-model estimates comparing Adaptive UF simulation with historically delivered treatment. Mean differences are reported with 95% confidence intervals. Models included a patient-specific random intercept to account for repeated treatments within patients. Patient-level and residual standard deviations and intraclass correlation coefficients (ICCs) are shown for each outcome. Positive mean differences indicate higher values during Adaptive UF simulation.

**Outcome**	**Mean difference (95% CI)**	**Patient-level SD**	**Residual SD**	**ICC**
Time within RBV target range (%)	20.2 (19.9–20.5)	13.3	28.8	0.18
Area outside RBV target range (RBV %-minutes)	−141.1 (−143.9 to −138.2)	139.2	194.6	0.34
Signed area outside RBV target range (RBV %-minutes)	−102.7 (−106.1 to −99.3)	173.4	209.4	0.41
Achieved UF volume (mL)	297.2 (293.5–300.8)	182.6	243.5	0.36
UF rate (mL/kg/h)	0.93 (0.91–0.94)	0.52	0.78	0.31
